# Racial, Ethnic, and Sex Differences in Methadone-Involved Overdose Deaths Before and After the US Federal Policy Change Expanding Take-home Methadone Doses

**DOI:** 10.1001/jamahealthforum.2023.1235

**Published:** 2023-06-09

**Authors:** Rebecca Arden Harris, Judith A. Long, Yuhua Bao, David S. Mandell

**Affiliations:** 1 Department of Family Medicine and Community Health, Perelman School of Medicine, University of Pennsylvania, Philadelphia; 2 Leonard Davis Institute of Health Economics, University of Pennsylvania, Philadelphia; 3 Corporal Michael J. Crescenz VA Medical Center, VA Center for Health Equity Research and Promotion, Philadelphia, Pennsylvania; 4 Division of General Internal Medicine, Perelman School of Medicine, University of Pennsylvania, Philadelphia; 5 Department of Population Health Sciences, Weill Cornell Medicine, New York, New York; 6 Department of Psychiatry, Perelman School of Medicine, University of Pennsylvania, Philadelphia

## Abstract

**Importance:**

In March 2020, the Substance Abuse and Mental Health Services Administration (SAMHSA) permitted states to relax restrictions on take-home methadone doses for treatment-adherent patients to minimize COVID-19 exposures.

**Objective:**

To assess whether the methadone take-home policy change was associated with drug overdose deaths among different racial, ethnic, and sex groups.

**Design, Setting, and Participants:**

Interrupted time series analysis from January 1, 2018, to June 30, 2022. Data analysis was conducted from February 18, 2023, to February 28, 2023. In this population-based cohort study of drug overdose mortality including 14 529 methadone-involved deaths, monthly counts of methadone-involved drug overdose deaths were obtained for 6 demographic groups: Hispanic men and women, non-Hispanic Black men and women, and non-Hispanic White men and women.

**Exposure:**

On March 16, 2020, in response to the first wave of the COVID-19 pandemic, SAMHSA issued an exemption to the states that permitted up to 28 days of take-home methadone for stable patients and 14 days for less stable patients.

**Main Outcome Measures:**

Monthly methadone-involved overdose deaths.

**Results:**

From January 1, 2018, to June 30, 2022 (54 months), there were 14 529 methadone-involved deaths in the United States; 14 112 (97.1%) occurred in the study’s 6 demographic groups (Black men, 1234; Black women, 754; Hispanic men, 1061; Hispanic women, 520; White men, 5991; and White women, 4552). Among Black men, there was a decrease in monthly methadone deaths associated with the March 2020 policy change (change of slope from the preintervention period, −0.55 [95% CI, −0.95 to −0.15]). Hispanic men also experienced a decrease in monthly methadone deaths associated with the policy change (−0.42 [95% CI, −0.68 to −0.17]). Among Black women, Hispanic women, White men, and White women, the policy change was not associated with a change in monthly methadone deaths (Black women, −0.27 [95% CI, −1.13 to 0.59]; Hispanic women, 0.29 [95% CI, −0.46 to 1.04]; White men, –0.08 [95% CI, −1.05 to 0.88]; and White women, −0.43 [95% CI, −1.26 to 0.40]).

**Conclusions and Relevance:**

In this interrupted time series study of monthly methadone-involved overdose deaths, the take-home policy may have helped reduce deaths for Black and Hispanic men but had no association with deaths of Black or Hispanic women or White men or women.

## Key Points

**Question:**

What has been the association between relaxing restrictions on take-home methadone doses and the number of fatal overdoses involving methadone among different racial, ethnic, and sex groups?

**Findings:**

In this interrupted time series cohort study of 14 529 methadone-involved overdose deaths, the methadone take-home policy was associated with reduced deaths among Black and Hispanic men but did not affect deaths of Black or Hispanic women or White men or women.

**Meaning:**

The urgency of the overdose crisis requires that national methadone policy debates and decisions address the heterogeneity of people in treatment; relaxing methadone restrictions may help some particularly at-risk groups.

## Teaser

This cohort study investigates racial, ethnic, and sex differences in methadone-involved overdose deaths in the United States before and after a federal policy change expanding take-home methadone doses.

## Introduction

For the past 50 years, outpatient methadone treatment for opioid use disorder has been tightly governed by federal statutes and regulations, which allow only federally certified opioid treatment programs (OTPs) to dispense methadone.^1^ Before the COVID-19 pandemic, most patients receiving methadone were required to present in person for initial evaluations and then in person 5 to 6 days per week for observed methadone dispensing of single doses. On March 16, 2020, in response to the first wave of the pandemic, the Substance Abuse and Mental Health Services Administration (SAMHSA) issued an exemption that allowed OTPs, with state approval, to provide up to 28 days of take-home methadone for patients who were stable and 14 days for those who were less stable.^2^ When taken as prescribed, methadone is safe and effective; however, there is a substantial risk of overdose when more than the amount prescribed is taken or when illicit opioids, such as heroin or fentanyl, are used in addition to methadone.^3^

The goal of the SAMHSA policy change was to protect public health by lowering the risk of COVID-19 infections among patients and health care clinicians (ie, accommodate social distancing while supporting patient care for treatment of opioid use disorder). Results of research on the effects of the policy change are still emerging, but the weight of the evidence suggests that the policy change was associated with an increase in the number of patients receiving take-home doses and that it did not adversely affect treatment outcomes, including fatal overdoses.^4,5,6,7,8,9,10,11,12^ A set of proposals that will permanently ease the take-home restrictions is now being considered by SAMHSA.^13^ To our knowledge, only 1 study has investigated the association between relaxing methadone restrictions and fatal overdoses among different racial, ethnic, or sex groups,^14^ although prior research suggests a strong possibility of demographic differences in outcomes. Research has shown the following: (1) Medication options available to persons with opioid use disorder differ by neighborhood, with OTPs dispensing methadone more often located in poor and minority neighborhoods, whereas less restrictive buprenorphine treatment services are more often located in higher-income areas with a higher percentage of White residents.^15,16^ (2) Access to OTP services differs by sex and race, with women and Black individuals spending more days on waiting lists to enter methadone treatment.^17^ (3) There are logistic impediments to retention in methadone treatment for women with children and family responsibilities.^18^ (4) There are lower methadone dosage regimens (“underdosing”) for Black patients and pregnant individuals from racial and ethnic minority groups.^19,20^

Reducing barriers to methadone may have a disproportionately positive effect on these groups.^21,22^

In the present study, we compared methadone-involved overdose deaths before and after the March 2020 policy change among Black, Hispanic, and White male and female individuals. Collectively, the 6 groups experienced 14 112 of the 14 529 fatal methadone-involved drug overdoses (97.1%).

## Methods

### Data Sources and Study Population

In this cohort study with interrupted time series analysis, we used US Centers for Disease Control and Prevention Wide-Ranging Online Data for Epidemiologic Research (2018 to 2021 final data and 2022 [January to June] provisional mortality data); data are from the multiple cause of death files as compiled from data provided by the 57 vital statistics jurisdictions through the Vital Statistics Cooperative Program.^23^ Drug overdose deaths had *International Statistical Classification of Diseases and Related Health Problems, Tenth Revision* (*ICD-10*) codes X40 to X44, X60 to X64, X85, and Y10 to Y14 for underlying cause of death; methadone-involved deaths had *ICD-10* code T40.3. The cause-of-death determination is typically made by a coroner or medical examiner.^24^

We extracted monthly drug overdose deaths that involved methadone from January 1, 2018, to June 30, 2022, for 6 demographic groups: Hispanic men and women (racial categories Black and White), non-Hispanic Black men and women, and non-Hispanic White men and women (eAppendix 1 in Supplement 1). The death certificate data on age, sex, race, and ethnicity are usually supplied by a funeral director, often as informed by the next of kin.^25^

We used interrupted time series analysis (ITSA) to model trends in monthly drug overdose deaths for each demographic group^26^ (eAppendix 1 in Supplement 1 for model specifications). A significant difference in the slopes before and after the take-home policy change would suggest that the policy change was associated with either an increase or decrease in methadone-related overdose deaths. With the 2022 ITSA study by Jones et al as our basis,^8^ we hypothesized that the policy change would not be associated with changes in slope. We also anticipated an implementation lag time of 4 to 6 weeks, even for the “early adopters” of the policy change. Because our postintervention time series ran 27 months, this lag time would not obscure the longer-term changes in the trend lines of methadone-involved overdose deaths. We did not have hypotheses regarding differences in the slope changes among the demographic groups. Therefore, our findings with respect to racial, ethnic, and sex groups should be considered exploratory.^27^

Given the expanding role of fentanyl in the overdose crisis (eFigure 2 in Supplement 1),^28^ we conducted a stratified analysis to control for the co-occurrence of synthetic opioids, mainly fentanyl and its analogs. Our aim was to determine whether the associations between the take-home policy and fatal methadone overdose were modified when separately estimated for (1) deaths that involved methadone but not fentanyl and (2) deaths that involved both methadone and fentanyl.

We also assessed whether monthly nonmethadone overdose deaths could provide a secular trend comparison, which would help assess whether a change in the trend line of methadone-involved deaths was associated with the take-home policy change or could be attributed to other factors affecting trends in drug overdose deaths generally. Nonmethadone deaths satisfied the 2 a priori criteria for a secular trend variable: a theory-based association between methadone deaths (ie, methadone and nonmethadone overdose deaths may be subject to the same broader social forces, although the populations may differ in some respects) and the absence of a theory-based association with the policy change (ie, trends in nonmethadone overdose deaths are not dependent on a change in the methadone take-home policy). A third criterion was strictly empirical: were methadone and nonmethadone overdose deaths closely or moderately correlated before the policy change? If not correlated in the preintervention period, then nonmethadone overdose deaths could not provide a basis for a postintervention comparison. Recent studies have used nonmethadone overdose deaths for secular trend purposes, but without providing empirical justification.^8,14^

Because this study used publicly available, deidentified data, the University of Pennsylvania institutional review board determined that approval and informed consent were not needed. The study followed the Strengthening the Reporting of Observational Studies in Epidemiology (STROBE) reporting guideline.

### Statistical Analysis

We used totals and percentages to describe overdose mortality by demographic category and mean values to summarize observations per data point. We used the Stata module ITSA to estimate the preintervention and postintervention slopes, the change in mortality levels, and the change in slopes. To adjust for autocorrelation, we used the Akaike information criterion for the lag order selection. The ITSA specifies the maximum lag to be considered in the autocorrelation structure using Newey-West SEs.^26^ We used Spearman ρ to measure the preintervention secular trend correlations. We considered 2-sided *P* < .05 statistically significant and used Stata, version 17.0 (StataCorp LLC) for data analysis. We conducted the data analysis from February 18, 2023, to February 23, 2023.

## Results

From January 1, 2018, to June 30, 2022, there were 406 484 drug overdose deaths in the US; 388 783 (95.6%) occurred in the study’s 6 demographic groups (Black men, 48 184; Black women, 18 489; Hispanic men, 34 686; Hispanic women, 9616; White men, 183 996; and White women, 93 812). During this period, methadone-involved overdose deaths totaled 14 529, or 3.6% of all drug overdose deaths. Of the methadone-involved deaths, 14 112 (97.1%) occurred in the study’s 6 demographic groups (Black men, 1234; Black women, 754; Hispanic men, 1061; Hispanic women, 520; White men, 5991; and White women, 4552).

### US Total

Before April 2020, methadone-involved overdose deaths were decreasing (preintervention slope, −1.36 [95% CI, −2.68 to −0.51]). In April 2020, methadone-involved overdose deaths increased by 115.18 deaths (95% CI, 79.85-150.51 deaths), estimated by subtracting the end point of the preintervention trend line from the starting point of the postintervention trend line. Monthly methadone-involved overdose deaths continued to decrease after April 2020 (postintervention slope, −3.07 [95% CI, −4.71 to –1.44]). The difference between the slopes before and after April 2020 was –1.71 deaths per month (95% CI, −3.84 to 0.42 deaths per month) (Figure, A; Table).

**Figure.**
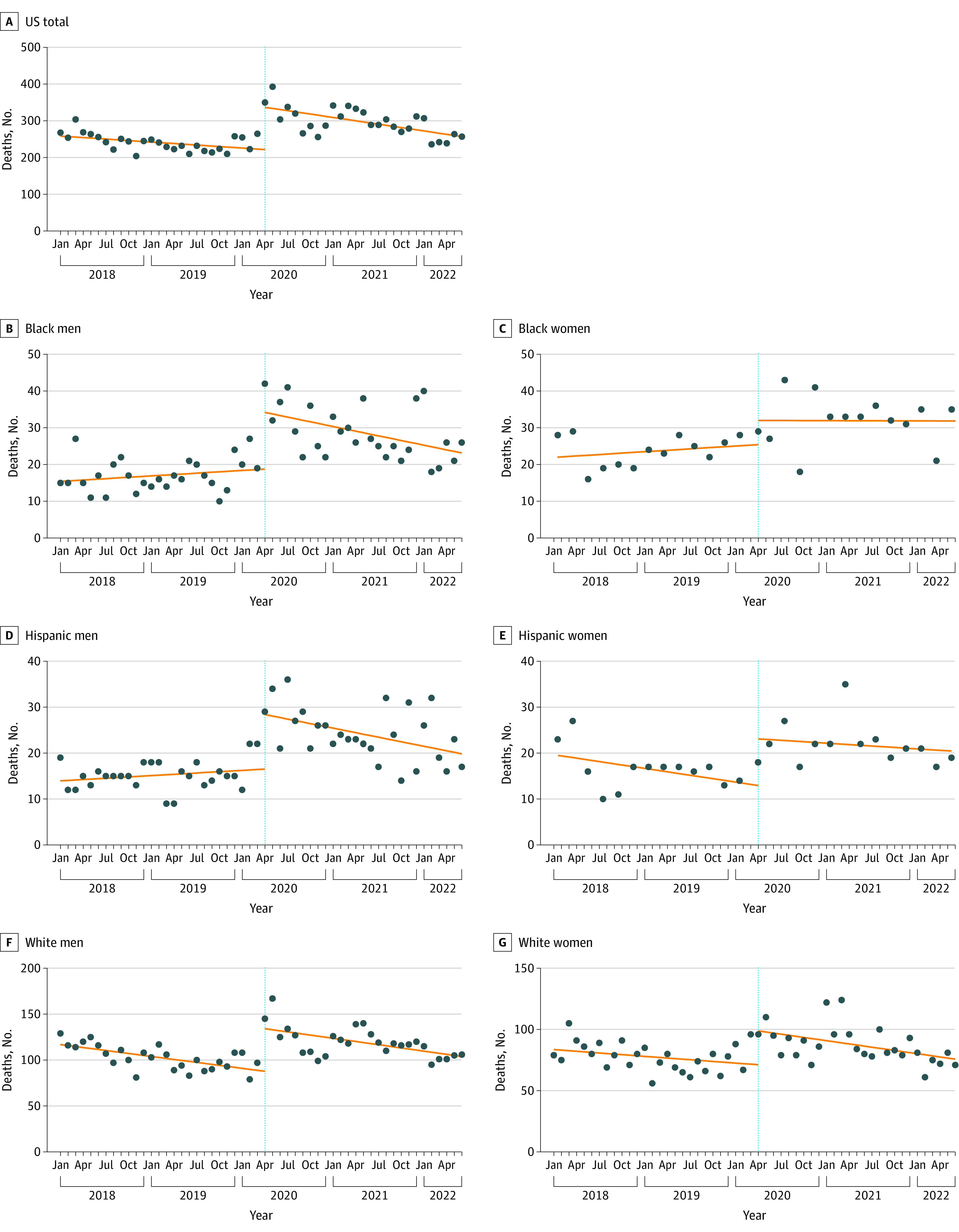
Monthly Methadone Overdose Deaths by Demographic Group and Year The circles are actual deaths, the orange trend lines are interrupted time series estimates, and the dotted vertical line indicates the start of the take-home policy. Because of the limited number of observations per month, estimates for Black and Hispanic women were based on bimonthly data (n = 27 rather than n = 54).

**Table.** Interrupted Times Series Analysis Estimates for Monthly Overdose Deaths Involving Methadone, January 2018 to June 2022

Parameter	Estimate (95% CI)	*P* value
**US total**		
Monthly trend (slope) before the take-home policy change	−1.36 (−2.68 to −0.51)	.04
Change in number of overdose deaths at time of policy change	115.18 (79.85 to 150.51)	<.001
Monthly trend (slope) after the policy change	−3.07 (−4.71 to −1.44)	<.001
Difference between slopes (before minus after)	−1.71 (−3.84 to 0.42)	.11
**Black men**		
Monthly trend (slope) before the take-home policy change	0.12 (−0.13 to 0.37)	.33
Change in number of overdose deaths at time of policy change	15.41 (9.69 to 21.13)	<.001
Monthly trend (slope) after the policy change	−0.42 (−0.71 to −0.14)	.004
Difference between slopes (before minus after)	−0.55 (−0.95 to −0.15)	.008
**Black women^a^**		
Monthly trend (slope) before the take-home policy change	0.26 (−0.45 to 0.97)	.46
Change in number of overdose deaths at time of policy change	6.58 (0.44 to 12.71)	.04
Monthly trend (slope) after the policy change	−0.01 (−0.57 to 0.54)	.97
Difference between slopes (before minus after)	−0.27 (−1.13 to 0.59)	.52
**Hispanic men**		
Monthly trend (slope) before the take-home policy change	0.09 (−0.06 to 0.25)	.23
Change in number of overdose deaths at time of policy change	11.88 (8.54 to 15.22)	<.001
Monthly trend (slope) after the policy change	−0.33 (−0.51 to −0.15)	.001
Difference between slopes (before minus after)	−0.42 (−0.68 to −0.17)	.002
**Hispanic women^a^**		
Monthly trend (slope) before the take-home policy change	−0.49 (−1.11 to 0.12)	.11
Change in number of overdose deaths at time of policy change	10.04 (4.63 to 15.44)	.001
Monthly trend (slope) after the policy change	−0.20 (−0.66 to 0.25)	.37
Difference between slopes (before minus after)	0.29 (−0.46 to 1.04)	.43
**White men**		
Monthly trend (slope) before the take-home policy change	−1.08 (−1.59 to −0.57)	<.001
Change in number of overdose deaths at time of policy change	46.46 (29.04 to 63.89)	<.001
Monthly trend (slope) after the policy change	−1.17 (−1.97 to −0.36)	.005
Difference between slopes (before minus after)	−0.08 (−1.05 to 0.88)	.86
**White women**		
Monthly trend (slope) before the take-home policy change	−0.46 (−1.08 to 0.16)	.14
Change in number of overdose deaths at time of policy change	27.64 (12.71 to 42.57)	.001
Monthly trend (slope) after the policy change	−0.89 (−1.41 to −0.36)	.001
Difference between slopes (before minus after)	−0.43 (−1.26 to 0.40)	.31

^a^ Because of the limited number of observations per month, estimates for Black and Hispanic women were based on bimonthly data (n = 27 rather than n = 54).

### Black Men

Before April 2020, ITSA-estimated monthly methadone-involved overdose deaths were stable; that is, there was no significant monthly increase or decrease (preintervention slope, 0.12 [95% CI, −0.13 to 0.37]). In April 2020, methadone-involved overdose deaths increased by 15.41 deaths (95% CI, 9.69-21.13 deaths). Monthly methadone-involved overdose deaths decreased after April 2020 (postintervention slope, −0.42 [95% CI, −0.71 to −0.14]). The difference between the slopes before and after April 2020 was significant (−0.55 [95% CI, −0.95 to −0.15]) (Figure, B; Table).

### Black Women

Before April 2020, methadone-involved overdose deaths were stable (preintervention slope, 0.26 [95% CI, −0.45 to 0.97]). In April 2020, methadone-involved overdose deaths increased by 6.58 deaths (95% CI, 0.44-12.71 deaths). Monthly methadone-involved overdose deaths remained stable after April 2020 (postintervention slope, −0.01 [95% CI, −0.57 to 0.54]). The before and after April 2020 slopes did not differ significantly (−0.27 [95% CI, −1.13 to 0.59]) (Figure, C; Table).

### Hispanic Men

Before April 2020, methadone-involved overdose deaths were stable (preintervention slope, 0.09 [95% CI, −0.06 to 0.25]). In April 2020, methadone-involved overdose deaths increased by 11.88 deaths (95% CI, 8.54-15.22 deaths). Monthly methadone-involved overdose deaths decreased after April 2020 (postintervention slope, −0.33 [95% CI, −0.51 to −0.15]). The before and after April 2020 slopes differed significantly (−0.42 [95% CI, −0.68 to −0.17]) (Figure, D; Table).

### Hispanic Women

Before April 2020, methadone-involved overdose deaths were decreasing slightly (preintervention slope, –0.49 [95% CI, −1.11 to 0.12]). In April 2020, methadone-involved overdose deaths increased by 10.04 deaths (95% CI, 4.63-15.44 deaths). Monthly methadone-involved overdose deaths were stable after April 2020 (postintervention slope, −0.20 [95% CI, −0.66 to 0.25]). The before and after April 2020 slopes did not differ significantly (0.29 [95% CI, −0.46 to 1.04]) (Figure, E; Table).

### White Men

Before April 2020, methadone-involved overdose deaths were decreasing (preintervention slope, −1.08 [95% CI, −1.59 to −0.57]). In April 2020, methadone-involved overdose deaths increased by 46.46 deaths (95% CI, 29.04-63.89 deaths). Monthly methadone-involved overdose deaths continued to decrease after April 2020 (postintervention slope, −1.17 [95% CI, −1.97 to –0.36]). The before and after April 2020 slopes were similar (–0.08 [95% CI, −1.05 to 0.88]) (Figure, F; Table).

### White Women

Before April 2020, methadone-involved overdose deaths were stable (preintervention slope, −0.46 [95% CI, −1.08 to 0.16]). In April 2020, methadone-involved overdose deaths increased by 27.64 deaths (95% CI, 12.71-42.57 deaths). Monthly methadone-involved overdose deaths decreased after April 2020 (postintervention slope, −0.89 [95% CI, −1.41 to –0.36]). The before and after April 2020 slopes were similar (–0.43 [95% CI, −1.26 to 0.40]) (Figure, G; Table). The regression estimates are summarized in the Table.

### Stratification by Coinvolvement of Synthetic Opioids (Mostly Fentanyl)

The results of the fentanyl stratification analysis are presented in the eTable, eFigure 1, and eAppendix 2 in Supplement 1. As with the unstratified analysis, only Black and Hispanic men had a significant change in the slope of monthly overdose deaths, both in the subset of overdose deaths that coinvolved fentanyl (−0.52 [95% CI, −0.94 to –0.10]) and the subset that did not (−0.45 [95% CI, −0.80 to –0.10]). Therefore, the decrease in slope after the policy change cannot be attributed to fentanyl coinvolvement.

### Secular Trend Analysis

In the secular trend analysis, monthly nonmethadone overdose deaths were uncorrelated with monthly methadone overdose deaths during the preintervention period in each of the 6 demographic groups and overall (Black men: Spearman *r* = 0.28, *P* = .15; Black women: *r* = 0.09, *P* = .66; Hispanic men: *r* = 0.09, *P* = .65; Hispanic women: *r* = –0.31, *P* = .11; White men: *r* = –0.25, *P* = .20; White women: *r* = 0.33, *P* = .09; and all persons: *r* = –0.13, *P* = .53).

## Discussion

Methadone-related deaths spiked in the US during the first wave of the COVID-19 pandemic^29,30^ in spring 2020 and then gradually decreased during the next 2 years; SAMHSA eased the restrictions on take-home doses as the first wave of the pandemic was starting to peak. For Black women, Hispanic women, White men, and White women, the postintervention trend lines were a continuation of the preintervention trends. However, there was an abrupt change for Black and Hispanic men. The slightly increasing number of methadone-involved deaths per month before the policy change became a sharply decreasing slope afterward (Figure), which suggests that the take-home policy may have particularly benefited these minoritized groups.

The overall decrease in methadone-involved drug deaths after the intervention does not appear to have been a function of fewer people taking methadone. In their study of buprenorphine and methadone supplies in the US, Chen and colleagues^31^ observed no substitution of buprenorphine treatment for methadone treatment during either the preintervention or postintervention period. Moreover, annual OTP orders for methadone, measured in grams, were relatively stable during the 4-year study period (2018, 11 368 111 g; 2019, 13 114 262 g; 2020, 13 116 235 g; 2021, 12 367 610 g; and January to June 2022, 6 154 552 g).^32^ But this is piecemeal evidence. Constructing a precise denominator for a methadone treatment overdose rate (eg, person-days of methadone use in OTPs) would require standardized national record keeping on the number of persons taking methadone for opioid use disorder and their duration of treatment.

In a stratification analysis, we estimated whether the associations between the take-home policy and fatal methadone overdose were modified when separately estimated for (1) deaths that involved methadone but not fentanyl and (2) deaths that involved methadone and fentanyl. We found that only Black and Hispanic men had a significant change (decrease) in the slope of monthly overdose deaths under both conditions. Thus, the decrease in slope after the policy change among Black and Hispanic men cannot be attributed to fentanyl. We also found that the preintervention slopes of monthly methadone deaths were positive when fentanyl was involved and negative when it was not. Similarly, each of the postintervention slopes was more positive when fentanyl was involved compared with the stratum without fentanyl. This pattern suggests that fentanyl was associated with an increase in the number of methadone-involved deaths both before and after the policy change across all demographic groups, but it does not appreciably strengthen, mitigate, or make conditional the associations (or lack of associations) between the policy change and methadone-involved overdose deaths.

The present study addressed several notable weaknesses in previous research. Jones et al,^8^ using ITSA to model the percentage of all drug overdose deaths that involved methadone, found that the trend slopes were similar before and after March 2020 and concluded that the evidence was consistent with permanently expanding the take-home methadone policy. However, their study did not empirically justify the use of nonmethadone overdose deaths as a secular trend variable (in a reanalysis of their time series, we found no correlation between nonmethadone and methadone overdose deaths during the preintervention period: Spearman *r* = 0.08; *P* = .79). Their study also did not stratify by demographic group, which effectively masked important differences in slope changes. In contrast to the results of Jones et al,^8^ Kleinman and Sanches^14^ reported that methadone-involved overdose deaths increased after the policy change, prompting the authors to warn against permanently relaxing the take-home regulations until the impact of the policy has been better clarified. In examining racial and ethnic differences in methadone-involved mortality, Kleinman and Sanches^14^ found that the number of individuals with methadone-involved deaths increased in each group (overall by 39%) between the 12-month periods before and after March 2020, with the largest increases among Hispanic (70%) and non-Hispanic Black individuals (57%). However, when assessing trends, their year-over-year analysis did not control for the step increase in mortality level that occurred in the early spring of 2020. The authors assumed that the step increase could be attributed to the change in methadone regulations, yet the increase in level also has been found in drug overdose deaths that did not involve methadone^8,33,34,35^ and in related domains, such as alcohol use disorder–associated deaths.^36,37,38^ Disruptions related to COVID-19 provide a more intuitive and parsimonious explanation for the step increases.^39^

In summary, we hypothesized that the policy change would not be associated with methadone-related overdose deaths. Although this was true for Black, Hispanic, and White women and for White men, we found fewer fatalities in Black and Hispanic men than expected. We did not expect to find differential associations based on demographic group and can only conjecture on the reason for these results: that having to daily report to an OTP is a distressing and demeaning experience for Black and Hispanic men who are already marginalized and continually exposed to systems of surveillance, stigma, alienation, and punishment. The additional take-home doses provided a sense of normalcy and dignity that was missing with frequent attendance at the OTP.^40^

### Limitations

This study has limitations. First, the lack of an external comparison, either a control group (persons taking methadone for opioid use disorder who were not exposed to the policy change) or a secular trend variable, precludes the drawing of causal inferences from the study data.^41^ As noted, the OTP take-home policy change happened in the context of concurrent trends (eg, unemployment and social isolation due to COVID-19), economic policy changes (eg, extended unemployment compensation, economic stimulus checks, and eviction moratoriums),^38^ and other policy changes (eg, increased use of telemedicine) that could have affected drug use and treatment for people with opioid use disorder. Second, analytic insight would be gained by relating the number of methadone-involved overdose deaths to the number of persons in methadone treatment each month by demographic group, but these data or reasonable proxies are not publicly available. Third, this study could not distinguish whether the individuals who died from methadone-involved overdoses received the methadone through OTPs (approximately 90% of all methadone supplies in the US are distributed to OTPs^32^), from pharmacy-dispensed prescriptions for pain (approximately 9%), or from other sources, including diverted methadone. Fourth, approximately 5% of death certificates do not list the specific drugs involved in the overdose.^8^ Fifth, the 2022 provisional mortality data may minimally underestimate overdose deaths because of delayed reporting.^42^

## Conclusions

In this interrupted time series cohort study of monthly methadone-involved overdose deaths, the take-home policy was associated with reduced deaths for Black and Hispanic men. However, no significant association was observed for Black or Hispanic women or White men or women. Regardless of the reason, the changes in slopes clearly display an association with race and ethnicity. The urgency of the drug overdose crisis requires that our national methadone policy debates and decisions attend to the heterogeneity of people in treatment.

## Notes

Supplement 1. **eAppendix 1.** eMethods. **eAppendix 2.** Stratification by Synthetic Opioids (Primarily Fentanyl and Analogs). **eTable.** Interrupted Times Series Analysis Estimates of the Difference Between Slopes in Monthly Methadone-Involved Overdose Deaths Before and After the Take Home Policy Change, Stratified by Synthetic Opioids (Mostly Fentanyl), January 2018 – June 2022 **eFigure 1.** Difference Between Slopes in Monthly Methadone-Involved Overdose Deaths Before and After the Take Home Policy Change, Stratified by Synthetic Opioids (Mostly Fentanyl and Analogs), January 2018 – June 2022 **eFigure 2.** Percent of Methadone-Involved Deaths That Also Involved Synthetic Opioids (Mostly Fentanyl and Analogs).
eReferences.

Supplement 2.
Data Sharing Statement
