# Supplementary material for: Racial, Ethnic, and Sex Differences in Methadone-Involved Overdose Deaths Before and After the US Federal Policy Change Expanding Take-home Methadone Doses
Source: JAMA Health Forum. 2023 Jun 9;4(6):e231235. doi: 10.1001/jamahealthforum.2023.1235 (PMC10257097; doi:10.1001/jamahealthforum.2023.1235)
Supplement: Supplement 1. — eAppendix 1. eMethods. eAppendix 2. Stratification by Synthetic Opioids (Primarily Fentanyl and Analogs). eTable. Interrupted Times Series Analysis Estimates of the Difference Between Slopes in Monthly Methadone-Involved Overdose Deaths Before and After the Take Home Policy Change, Stratified by Synthetic Opioids (Mostly Fentanyl), January 2018 – June 2022 eFigure 1. Difference Between Slopes in Monthly Methadone-Involved Overdose Deaths Before and After the Take Home Policy Change, Stratified by Synthetic Opioids (Mostly Fentanyl and Analogs), January 2018 – June 2022 eFigure 2. Percent of Methadone-Involved Deaths That Also Involved Synthetic Opioids (Mostly Fentanyl and Analogs). eReferences. [file jamahealthforum-e231235-s001.pdf]

Supplementary Online Content

Harris RA, Long JA, Bao Y, Mandell DS. Racial, ethnic, and sex differences in methadone-involved overdose deaths before and after the US federal policy change expanding take-home methadone doses. *JAMA Health Forum.* 2023;4(6):e231235. doi:10.1001/jamahealthforum.2023.1235

eAppendix 1. eMethods.
eAppendix 2. Stratification by Synthetic Opioids (Primarily Fentanyl and Analogs).
eTable. Interrupted Times Series Analysis Estimates of the Difference Between Slopes in Monthly Methadone-Involved Overdose Deaths Before and After the Take Home Policy Change, Stratified by Synthetic Opioids (Mostly Fentanyl), January 2018 – June 2022
eFigure 1. Difference Between Slopes in Monthly Methadone-Involved Overdose Deaths Before and After the Take Home Policy Change, Stratified by Synthetic Opioids (Mostly Fentanyl and Analogs), January 2018 – June 2022
eFigure 2. Percent of Methadone-Involved Deaths That Also Involved Synthetic Opioids (Mostly Fentanyl and Analogs).
eReferences.

This supplemental material has been provided by the authors to give readers additional information about their work.

eAppendix 1. eMethods

1. Selection of demographic groups based on observations per time point

The six racial, ethnic, and gender groups were chosen because they each averaged ≥15 methadone−involved deaths per time point over the 54-month study period (Black men: 22.9; Black women: 27.9; Hispanic men: 19.6; Hispanic women: 19.3; White men: 110.9; White women: 84.3). Each month was considered a time point for Black men, Hispanic men, White men, and White women. To reach an average of 15 and lower the risk of Type II error, we combined adjacent time points (2 months per time point) for Black and Hispanic women, reducing the number of time points in the analysis from 54 to 27. Though halving the number of time points marginally decreased statistical power, 27 time points is sufficient for interrupted time series analysis.[1] American Indian, Alaska Native, Asian, Native Hawaiian, and Other Pacific Islander individuals were excluded from this study because they had too few observations per time point to analyze.

2. Interrupted time series analysis (ITSA) regression model

The standard interrupted time series regression model takes the form:

$$Y_t = B_0 + B_1 T_t + B_2 X_t + B_3 X_t T_t + e_t$$

where

Y_t is the outcome variable measured at each time point $_t$

T_t is the time since the start of the study

X_t is a dummy variable representing the intervention (pre-intervention periods 0, otherwise 1)

$X_t T_t$ is an interaction term

B_0 is the intercept

B_1 is the slope prior to the intervention

B_2 is the change in the level of the outcome variable immediately following the introduction of the intervention (compared to the counterfactual)

B_3 is the difference between the pre- and post-intervention slopes of the outcome, and

e_t is the error term.

We used ITSA is to assess whether there was a change in the level or trend of methadone-involved deaths following the SAMHSA take-home policy change. The model constructs a counterfactual – what the trend would look like in the absence of the policy change – which is then compared with the post-intervention actual trend.

eAppendix 2.

1. Stratification by synthetic opioids (primarily fentanyl and analogs).

The Figure and Table in the Results section showed that the association between the take home policy change and monthly methadone-involved overdose deaths differed by demographic group. Specifically, monthly methadone-involved deaths decreased in the Black and Hispanic male groups after the policy change; no change in the before and after trend lines was observed in the other demographic groups.

We now stratify this analysis by the co-involvement of fentanyl. We used ICD-10 diagnostic category T40.4, which refers to poisoning by synthetic opioids (mostly fentanyl or fentanyl analogs), hereafter “fentanyl.” Our aim is to determine whether the associations between the take home policy and fatal methadone overdose are modified when separately estimated for (1) deaths that involved methadone but not fentanyl, and (2) deaths that involved both methadone and fentanyl. The results of the stratification analysis are presented in the eTable.

First, it should be noted that several demographic groups had very few observations per time point after stratification (column 3 in the eTable). By the standard of a minimum of 15 observations per time point, Black and Hispanic women did not have enough observations to reliably estimate ITSA parameters. The results for Black and Hispanic women should therefore be interpreted cautiously. The number of observations per time point also was low for Black and Hispanic men after stratification. Because Black and Hispanic men showed the same pattern of slopes (see Figure and Table in the Results section), and because they may have had OTP experiences in common as members of minoritized communities, we combined the groups to increase the number of observations per time point while preserving the 54 time points. Observations per time point and number of time points both contribute to power, along with effect size, a balanced number of study periods before and after an intervention, and other factors.[1,2]

Of the demographic groups, only Black and Hispanic men had a significant change (decrease) in the slope of monthly overdose deaths, both with the subset of overdose deaths that co-involved fentanyl (−0.52; 95% CI: −0.94 to –0.10) and the subset that did not (−0.45; 95% CI: −0.80 to –0.10). Thus, the decrease in slope following the policy change among Black and Hispanic men cannot be attributable to fentanyl.

Although fentanyl did not account for the before/after slope differences across the demographic groups, fentanyl did affect the direction of the pre-intervention slopes. eFigure 1 shows that the pre-intervention slopes of monthly methadone deaths were generally positive when fentanyl was involved and negative when fentanyl was not involved. Similarly, each of the post-intervention slopes were more positive when fentanyl was involved compared to the without-fentanyl stratum. This pattern suggests that fentanyl raised the number of both pre- and post-policy change methadone-involved deaths across all demographic groups, but did not appreciably strengthen, mitigate, or make conditional the associations (or lack of associations) between the policy change and methadone-involved overdose deaths.

eTable. Interrupted times series analysis estimates of the difference between slopes in monthly methadone-involved overdose deaths before and after the take home policy change, stratified by synthetic opioids (mostly fentanyl), January 2018 – June 2022

Parameter	Time Points	Mean Deaths per Time Pts	Estimate	(95% CI)	P−value
**United States total:**					
Before/After difference in slopes – with fentanyl	54	113.8	−0.71	(−2.05 to 0.63)	.292
Before/After difference in slopes – without fentanyl	54	155.3	−1.00	(−2.28 to 0.29)	.125
**Black men:**					
Before/After difference in slopes – with fentanyl	54	15.3	−0.31	(−0.64 to 0.02)	.069
Before/After difference in slopes – without fentanyl	54	7.5	−0.24	(−0.45 to −0.03)	.027
***Black women:**					
Before/After difference in slopes – with fentanyl	27	15.8	−0.42	(−0.89 to 0.06)	.085
Before/After difference in slopes – without fentanyl	27	12.1	0.15	(−0.54 to 0.83)	.663
**Hispanic men:**					
Before/After difference in slopes – with fentanyl	54	9.9	−0.21	(−0.40 to −0.03)	.022
Before/After difference in slopes – without fentanyl	54	9.8	−0.21	(−0.44 to 0.02)	.071
***Hispanic women:**					
Before/After difference in slopes – with fentanyl	27	7.9	−0.04	(−0.55 to 0.47)	.881
Before/After difference in slopes – without fentanyl	27	11.3	0.33	(−0.53 to 1.19)	.440
**White men:**					
Before/After difference in slopes – with fentanyl	54	46.4	0.10	(−0.45 to 0.64)	.723
Before/After difference in slopes – without fentanyl	54	64.5	−0.18	(−0.83 to 0.47)	.579
**White women:**					
Before/After difference in slopes – with fentanyl	54	26.7	–0.11	(–0.52 to 0.31)	.606
Before/After difference in slopes – without fentanyl	54	55.8	–0.32	(–0.93 to 0.29)	.299
**Black and Hispanic men combined:**					
Before/After difference in slopes – with fentanyl	54	25.2	−0.52	(−0.94 to –0.10)	.016
Before/After difference in slopes – without fentanyl	54	17.3	−0.45	(−0.80 to –0.10)	.014

*Because of the limited number of observations per month, estimates for Black and Hispanic women were based on bimonthly data (n=27 rather than n=54).

eFigure 1. Difference between slopes in monthly methadone-involved overdose deaths before and after the take home policy change, stratified by synthetic opioids (mostly fentanyl and analogs), January 2018 – June 2022

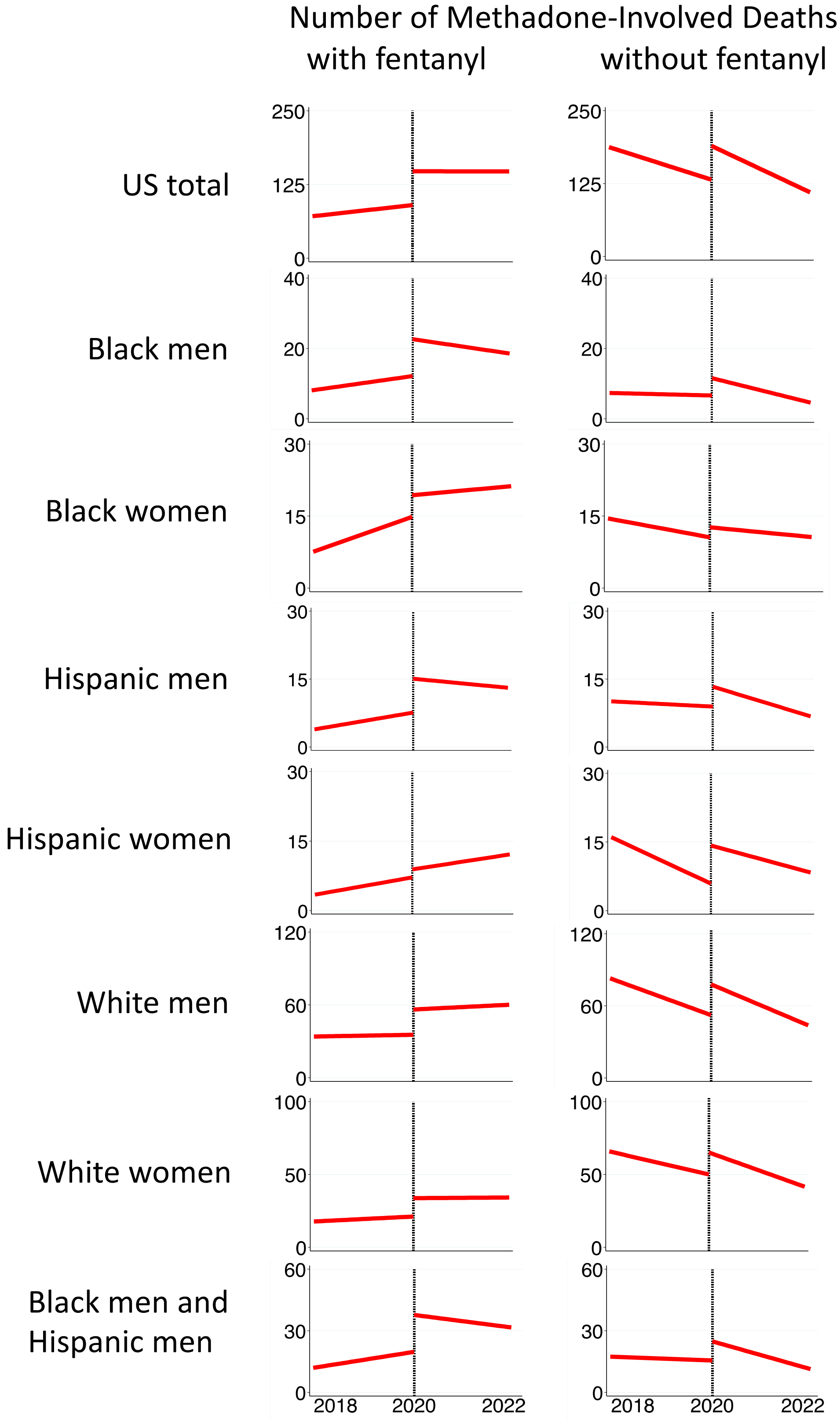

© 2023 Harris RA et al. *JAMA Health Forum.*

eFigure 2 shows the increasing role of fentanyl in methadone-involved overdose deaths during the study period (January 1, 2018 to June 30, 2022). Co-involvement of fentanyl increased in each demographic group. Black men and women had the highest percentages, both before and after the policy change. White women had the lowest percentage of fentanyl co-involvement.

eFigure 2. Percent of methadone-involved deaths that also involved synthetic opioids (mostly fentanyl and analogs).
Monthly deaths are presented in left panel (bimonthly for Black and Hispanic women). In the right panel, data are Lowess smoothed (running means).

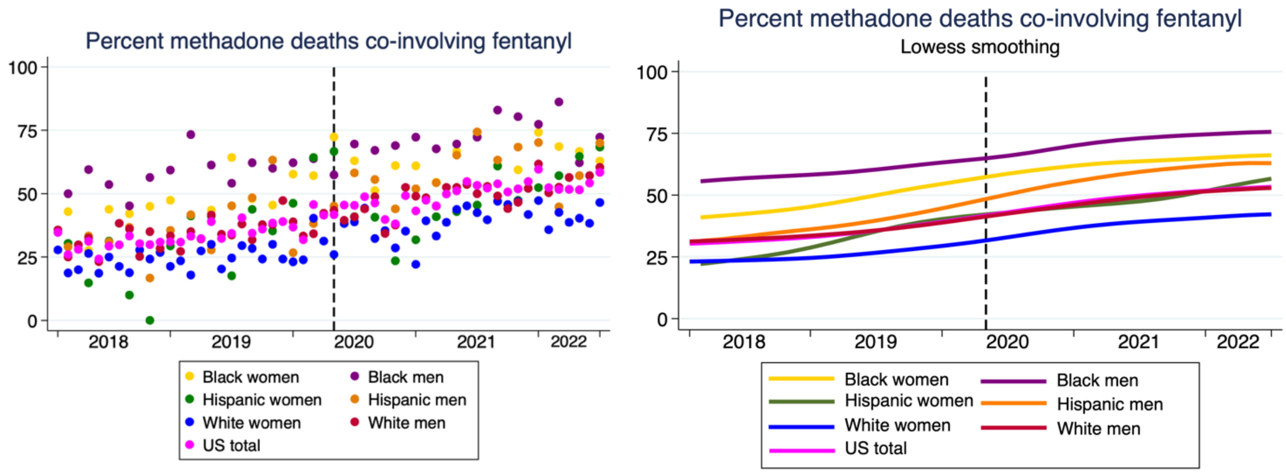

eReferences

1. Hawley S, Ali MS, Berencsi K, Judge A, Prieto−Alhambra D. Sample size and power considerations for ordinary least squares interrupted time series analysis: a simulation study. Clin Epidemiol. 2019 Feb 25;11:197−205. doi: 10.2147/CLEP.S176723. PMID: 30881136; PMCID: PMC6394245.

2. Zhang F, Wagner AK, Ross-Degnan D. Simulation-based power calculation for designing interrupted time series analyses of health policy interventions. J Clin Epidemiol. 2011;64(11):1252–1261.
