# Supplementary material for: Racial, Ethnic, and Sex Differences in Methadone-Involved Overdose Deaths Before and After the US Federal Policy Change Expanding Take-home Methadone Doses
Source: JAMA Health Forum. 2023 Jun 9;4(6):e231235. doi: 10.1001/jamahealthforum.2023.1235 (PMC10257097; doi:10.1001/jamahealthforum.2023.1235)
Supplement: Supplement 2. — Data Sharing Statement [file jamahealthforum-e231235-s002.pdf]

Data Sharing Statement

Harris. Racial, Ethnic, and Sex Differences in Methadone-Involved Overdose Deaths Before and After the US Federal Policy Change Expanding Take-Home Methadone Doses. *JAMA Health Forum*. Published June 09, 2023. doi:10.1001/jamahealthforum.2023.1235

Data
Data available: Yes
Data types: Deidentified participant data
How to access data: The data for this study were compiled from the publicly available Centers for Disease Control and Prevention, National Center for Health Statistics. National Vital Statistics System, CDC WONDER Online Database, which can be accessed at http://wonder.cdc.gov.
When available: With publication

Supporting Documents
Document types: None

Additional Information
Who can access the data: anyone
Types of analyses: any purpose
Mechanisms of data availability: No support or access agreement required.
Any additional restrictions: none
